# Bis[*N*-(2-amino­eth­yl)ethane-1,2-diamine-κ^3^ *N*,*N*′,*N*′′]copper(II) tris­[diamminetetra­kis­(thio­cyanato-κ*N*)chromate(III)] thio­cyanate dimethyl sulfoxide tetra­deca­solvate monohydrate

**DOI:** 10.1107/S1600536811025980

**Published:** 2011-07-06

**Authors:** Vitalina M. Nikitina, Oksana V. Nesterova, Roman I. Zubatyuk, Oleg V. Shishkin, Julia A. Rusanova

**Affiliations:** a Taras Shevchenko National University of Kyiv, Department of Inorganic Chemistry, Volodymyrska str. 64, Kyiv 01033, Ukraine; b Institute for Scintillation Materials, "Institute for Single Crystals", National Academy of Sciences of Ukraine, Lenina ave. 60, Kharkov 61001, Ukraine

## Abstract

The ionic title complex, [Cu(C_4_H_13_N_3_)_2_]_2_[Cr(NCS)_4_(NH_3_)_2_]_3_(NCS)·14C_2_H_6_OS·H_2_O, consists of complex [Cu(dien)_2_]^2+^ cations [dien is *N*-(2-amino­eth­yl)ethane-1,2-diamine], com­plex [Cr(NCS)_4_(NH_3_)_2_]^−^ anions, an NCS^−^ counter-anion and uncoordinated dimethyl sulfoxide (DMSO) and water solvent mol­ecules. One of the Cr atoms lies on an inversion center, while the second Cr atom and the Cu atom lie in general positions. The thio­cyanate counter-anion and water mol­ecule are disordered over two positions close to an inversion center. There are several types of hydrogen-bond inter­actions present in the title compound, which connect the complex cations and anions into bulky [Cu_2_Cr_3_] polynuclear species. The four NH_3_ groups of the complex anions and six bridging DMSO O atoms link the three complex anions *via* hydrogen bonding into the anionic polynuclear species [Cr(NCS)_4_(NH_3_)_2_]_3_·6DMSO. The last one is connected by four bridging DMSO O atoms with the two complex copper cations through N—H⋯ O hydrogen bonds between the terminal NH_3_ groups of the anionic polynuclear species and the NH and NH_2_ groups of the dien ligand. One additional DMSO mol­ecule is connected *via* hydrogen bonding to one of the terminal NH_3_ groups of the anionic polynuclear species. Another DMSO mol­ecule is connected *via* hydrogen bonding to each Cu(dien)_2_]^2+^ cation.

## Related literature

For background to direct synthesis, see: Nesterov *et al.* (2004 ▶, 2006 ▶); Kovbasyuk *et al.* (1997 ▶, 1998 ▶); Vassilyeva *et al.* (1997 ▶). For related stuctures, see: Zhang *et al.* (2001 ▶); Cucos *et al.* (2006 ▶); Cherkasova & Gorunova (2003 ▶); Kolotilov *et al.* (2010 ▶).
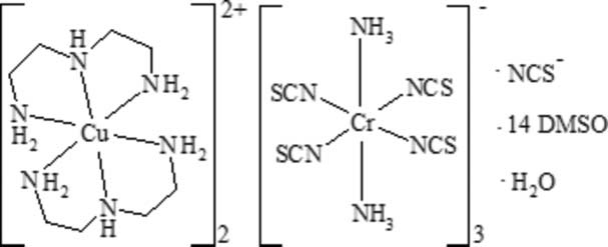


## Experimental

### 

#### Crystal data

[Cu(C_4_H_13_N_3_)_2_]_2_[Cr(NCS)_4_(NH_3_)_2_]_3_(NCS)·14C_2_H_6_OS·H_2_O
*M* *_r_* = 2665.12
Monoclinic, 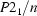

*a* = 11.9110 (2) Å
*b* = 26.5332 (5) Å
*c* = 20.2756 (4) Å
β = 91.256 (2)°
*V* = 6406.3 (2) Å^3^
*Z* = 2
Mo *K*α radiation
μ = 1.07 mm^−1^
*T* = 100 K
0.5 × 0.4 × 0.3 mm

#### Data collection

Oxford Diffraction Xcalibur Sapphire3 diffractometer
Absorption correction: multi-scan (*CrysAlis RED*; Oxford Diffraction, 2007) ▶ *T*_min_ = 0.60, *T*_max_ = 0.72
32460 measured reflections
14316 independent reflections
9143 reflections with *I* > 2σ(*I*)
*R*_int_ = 0.043

#### Refinement

*R*[*F* ^2^ > 2σ(*F* ^2^)] = 0.048
*wR*(*F* ^2^) = 0.127
*S* = 1.08
14316 reflections
660 parameters
H-atom parameters constrained
Δρ_max_ = 0.96 e Å^−3^
Δρ_min_ = −0.83 e Å^−3^

### 

Data collection: *CrysAlis PRO* (Oxford Diffraction, 2010 ▶); cell refinement: *CrysAlis PRO*; data reduction: *CrysAlis PRO*; program(s) used to solve structure: *SHELXS97* (Sheldrick, 2008 ▶); program(s) used to refine structure: *SHELXL97* (Sheldrick, 2008 ▶); molecular graphics: *SHELXTL* (Sheldrick, 2008 ▶); software used to prepare material for publication: *publCIF* (Westrip, 2010 ▶).

## Supplementary Material

Crystal structure: contains datablock(s) I, global. DOI: 10.1107/S1600536811025980/br2170sup1.cif

Structure factors: contains datablock(s) I. DOI: 10.1107/S1600536811025980/br2170Isup2.hkl

Additional supplementary materials:  crystallographic information; 3D view; checkCIF report

## Figures and Tables

**Table 1:** Hydrogen-bond geometry (Å, °)

*D*—H⋯*A*	*D*—H	H⋯*A*	*D*⋯*A*	*D*—H⋯*A*
O1*W*—H1*W*⋯N16	0.86	2.02	2.83 (3)	157
N1—H1*N*⋯O2	0.97	2.10	3.029 (4)	158
N2—H2*A*⋯O5	0.92	2.26	3.083 (4)	149
N2—H2*B*⋯O1*W*	0.92	2.09	2.919 (10)	150
N4—H4*N*⋯O7	0.99	1.96	2.928 (4)	167
N5—H5*A*⋯O5	0.92	2.24	2.996 (4)	140
N6—H6*B*⋯S14^i^	0.92	2.62	3.539 (6)	174
N9—H9*A*⋯O1	0.91	2.16	3.049 (3)	165
N9—H9*B*⋯O6	0.91	2.02	2.881 (4)	157
N9—H9*C*⋯O3	0.91	2.23	3.093 (4)	157
N14—H14*A*⋯O3	0.91	2.19	3.001 (4)	148
N14—H14*B*⋯O6	0.91	1.96	2.853 (4)	169
N14—H14*C*⋯O1	0.91	2.18	3.066 (4)	166
N15—H15*A*⋯O4	0.91	2.12	3.023 (4)	175
N15—H15*B*⋯O5	0.91	2.06	2.965 (4)	173
N15—H15*C*⋯O2	0.91	2.09	2.992 (4)	172
O1*W*—H1*W*⋯N16	0.86	2.02	2.83 (3)	157
N3—H3*A*⋯S4^ii^	0.92	2.67	3.518 (3)	154
N5—H5*B*⋯S1^iii^	0.92	2.66	3.529 (3)	158
O1*W*—H2*W*⋯S6^iv^	0.87	2.77	3.523 (11)	145
N6—H6*A*⋯S4^ii^	0.92	2.80	3.686 (4)	161

Symmetry codes: (i) 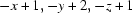; (ii) 
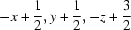
; (iii) 
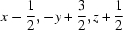
; (iv) 
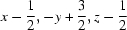
.

**Fig. 1.**
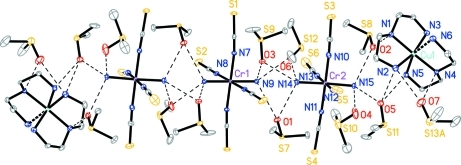
Molecular view of the title compound. Displacement ellipsoids are drawn at the 30% probability level.

**Fig. 2.**
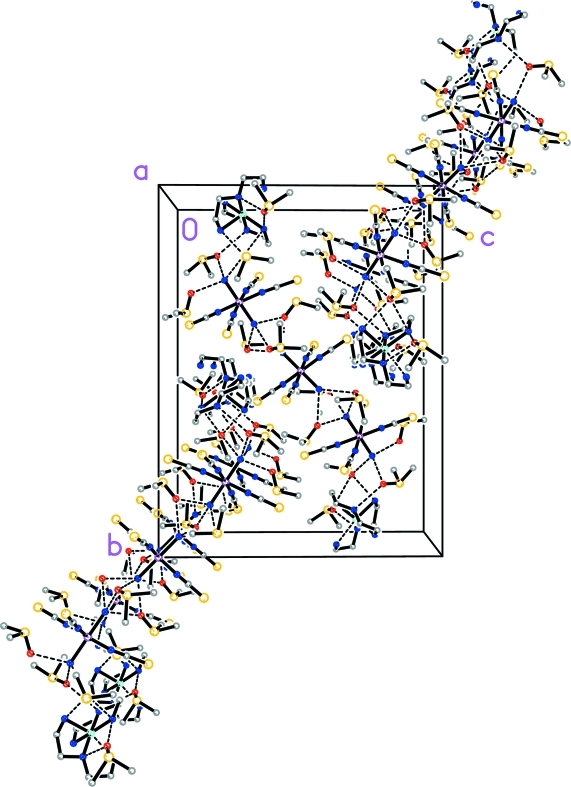
Crystal packing of the title compound along a axis.

## supplementary crystallographic information

### Comment

As it was shown in our previous work direct synthesis is an efficient method to obtained novel homo- and heterometallic complexes (Nesterov *et al.* (2004, 2006); Kovbasyuk *et al.* (1997, 1998); Vassilyeva *et al.* (1997)). Continuing our investigations in this paper we present a novel Cu/Cr heterometallic ionic complex which has been synthesized using zerovalent copper, Reinecke's salt and diethylenetriamine as starting materials.

As it shown on Fig.1 Cu atoms in complex cations are in distorted square bypiramidal coordination environment - four N atoms in equatorial position and two N atoms in axial position. The Cr centers are coordinated to six N atoms - four NCS-groups in equatorial position and two NH_3_ molecules in axial position.The four NH_3_ groups of the complex anion and bridging six oxygen atoms of solvent DMSO H-link the three complex anions into the anionic polynuclear species [Cr(NCS)_4_(NH_3_)_2_]_3_^.^ 6DMSO. The last one is connected by four bridging oxygen atoms of the solvent (DMSO) molecues with the two complex copper cations through N—H··· O hydrogen bonds between the terminal NH_3_ groups of the anionic polynuclear species and NH, NH_2_ groups of the dien ligand. One additional DMSO molecules is H-connected to one of the terminal NH_3_ groups of the anionic polynuclear species. Another one DMSO molecule is H-connected to each Cu(dien)_2_]^2+^ cations.

The bond distances and angles in the title molecule agree well with the corresponding bond distances and angles reported in closely related compounds (Zhang *et al.*, 2001, Cucos *et al.*, 2006; Cherkasova *et al.*, 2003; Kolotilov *et al.*, 2010). The crystal packing of the title compound is presented on Fig 2.

### Experimental

For the preparation of the title compound, copper powder 0.08 g (1.26 mmol), NH_4_[Cr(NCS)_4_(NH_3_)_2_]^.^H_2_O 0.10 g (1.26 mmol), NH_4_NCS 0,10 g (1,26 mmol), 0.27 ml (2.52 mmol) diethylenetriamine (dien), 20 ml of DMSO, were heated to 323–333 K and stirred magnetically for 15 min, until total dissolution of the copper powder was observed. Addition of a few ml of the Pr^i^OH to the cooled solution leads to precipitation within few days of the dark blue crystals suitable for X-ray analysis. They were collected by filter-suction, washed with dry Pr^i^OH and finally dried in *vacuo* at room temperature (yield: 0.89 g, 53%).

### Refinement

The NCS group lies close to the inversion center and refined with multiplicity 0.5. The water molecules refined with multiplicity of 0.5 because their displacement depends on the orientation of NCS anions. One of the DMSO molecules is disordered over two positions A and B for sulfur atoms (S13) and methyl group (C27) with multiplicity of 0.687 and 0.313 respectively. The second DMSO molecule is also disordered over two positions A and B with multiplicity of 0.65 and 0.35 for C23 and C24 atoms. All hydrogen atoms where placed at calculated positions which were refined as 'riding' model.

### Crystal data

**Table:**

[Cu(C_4_H_13_N_3_)_2_]_2_[Cr(NCS)_4_(NH_3_)_2_]_3_(NCS)·14C_2_H_6_OS·H_2_O	*F*(000) = 2794
*M**_r_* = 2665.12	*D*_x_ = 1.381 Mg m^−^^3^
Monoclinic, *P*2_1_/*n*	Mo *K*α radiation, λ = 0.71073 Å
Hall symbol: -P 2yn	Cell parameters from 6255 reflections
*a* = 11.9110 (2) Å	θ = 2.7–25.0°
*b* = 26.5332 (5) Å	µ = 1.07 mm^−^^1^
*c* = 20.2756 (4) Å	*T* = 100 K
β = 91.256 (2)°	Prism, dark-blue
*V* = 6406.3 (2) Å^3^	0.5 × 0.4 × 0.3 mm
*Z* = 2	

### Data collection

**Table:**

Oxford Diffraction Xcalibur Sapphire3 diffractometer	14316 independent reflections
Radiation source: fine-focus sealed tube	9143 reflections with *I* > 2σ(*I*)
graphite	*R*_int_ = 0.043
ω and φ scans	θ_max_ = 27.5°, θ_min_ = 2.7°
Absorption correction: multi-scan (*CrysAlis RED*; Oxford Diffraction, 2007)	*h* = −15→11
*T*_min_ = 0.60, *T*_max_ = 0.72	*k* = −34→33
32460 measured reflections	*l* = −25→26

### Refinement

**Table:**

Refinement on *F*^2^	Primary atom site location: structure-invariant direct methods
Least-squares matrix: full	Secondary atom site location: difference Fourier map
*R*[*F*^2^ > 2σ(*F*^2^)] = 0.048	Hydrogen site location: inferred from neighbouring sites
*wR*(*F*^2^) = 0.127	H-atom parameters constrained
*S* = 1.08	*w* = 1/[σ^2^(*F*_o_^2^) + (0.0585*P*)^2^] where *P* = (*F*_o_^2^ + 2*F*_c_^2^)/3
14316 reflections	(Δ/σ)_max_ = 0.092
660 parameters	Δρ_max_ = 0.96 e Å^−^^3^
0 restraints	Δρ_min_ = −0.83 e Å^−^^3^

### Special details

**Table:**

Experimental. CrysAlis RED, Oxford Diffraction Ltd., Version 1.171.32.5 (release 08-05-2007 CrysAlis171 .NET) (compiled May 8 2007,13:10:02) Empirical absorption correction using spherical harmonics, implemented in SCALE3 ABSPACK scaling algorithm.
Geometry. All e.s.d.'s (except the e.s.d. in the dihedral angle between two l.s. planes) are estimated using the full covariance matrix. The cell e.s.d.'s are taken into account individually in the estimation of e.s.d.'s in distances, angles and torsion angles; correlations between e.s.d.'s in cell parameters are only used when they are defined by crystal symmetry. An approximate (isotropic) treatment of cell e.s.d.'s is used for estimating e.s.d.'s involving l.s. planes.
Refinement. Refinement of *F*^2^ against ALL reflections. The weighted *R*-factor *wR* and goodness of fit *S* are based on *F*^2^, conventional *R*-factors *R* are based on *F*, with *F* set to zero for negative *F*^2^. The threshold expression of *F*^2^ > σ(*F*^2^) is used only for calculating *R*-factors(gt) *etc*. and is not relevant to the choice of reflections for refinement. *R*-factors based on *F*^2^ are statistically about twice as large as those based on *F*, and *R*- factors based on ALL data will be even larger.

### Fractional atomic coordinates and isotropic or equivalent isotropic displacement parameters (Å^2^)

**Table:**

	*x*	*y*	*z*	*U*_iso_*/*U*_eq_	Occ. (<1)
Cu1	0.30425 (3)	0.940916 (16)	0.71495 (2)	0.01954 (11)	
Cr1	0.5000	0.5000	0.5000	0.01345 (16)	
Cr2	0.33118 (5)	0.69446 (2)	0.72774 (3)	0.01687 (13)	
S1	0.84672 (8)	0.57936 (4)	0.44691 (5)	0.0271 (2)	
S2	0.70040 (9)	0.40980 (5)	0.66907 (5)	0.0416 (3)	
S3	0.66310 (9)	0.78060 (5)	0.66180 (6)	0.0403 (3)	
S4	−0.00734 (8)	0.59999 (4)	0.75949 (5)	0.0320 (2)	
S5	0.23280 (11)	0.75385 (5)	0.51398 (5)	0.0525 (4)	
S6	0.51532 (14)	0.63383 (6)	0.92336 (7)	0.0704 (5)	
S7	0.10788 (7)	0.56430 (3)	0.57327 (4)	0.01877 (18)	
S8	0.52484 (8)	0.82411 (3)	0.87334 (5)	0.0240 (2)	
S9	0.69825 (7)	0.57774 (4)	0.71490 (5)	0.0243 (2)	
S10	0.11920 (13)	0.65903 (4)	0.91109 (6)	0.0534 (4)	
S11	−0.01903 (8)	0.80693 (4)	0.69178 (5)	0.0337 (2)	
S12	0.53845 (11)	0.70282 (4)	0.51768 (6)	0.0426 (3)	
S13A	−0.1106 (3)	0.93726 (18)	0.6103 (2)	0.0593 (12)	0.687 (10)
S13B	−0.1000 (5)	0.9585 (3)	0.5913 (3)	0.0374 (16)	0.313 (10)
S14	0.5988 (6)	1.0131 (3)	0.5268 (3)	0.0742 (15)	0.50
O1	0.21600 (19)	0.56121 (10)	0.61363 (12)	0.0256 (6)	
O2	0.4432 (2)	0.83055 (10)	0.81576 (13)	0.0301 (6)	
O3	0.5771 (2)	0.56285 (10)	0.70545 (13)	0.0280 (6)	
O4	0.1377 (3)	0.71349 (12)	0.89450 (16)	0.0636 (11)	
O5	0.1042 (2)	0.81691 (10)	0.70353 (14)	0.0335 (7)	
O6	0.5166 (3)	0.65800 (10)	0.56202 (15)	0.0538 (10)	
O7	0.0099 (3)	0.92967 (16)	0.62847 (19)	0.0708 (12)	
N1	0.4518 (2)	0.90523 (12)	0.70298 (15)	0.0256 (7)	
H1N	0.4588	0.8757	0.7311	0.031*	
N2	0.2500 (3)	0.89679 (12)	0.63576 (16)	0.0309 (8)	
H2A	0.1861	0.8794	0.6464	0.037*	
H2B	0.2341	0.9167	0.5996	0.037*	
N3	0.3992 (2)	0.98174 (12)	0.78516 (15)	0.0255 (7)	
H3A	0.4018	1.0152	0.7732	0.031*	
H3B	0.3662	0.9796	0.8257	0.031*	
N4	0.1596 (2)	0.97955 (10)	0.72457 (13)	0.0161 (6)	
H4N	0.0999	0.9646	0.6964	0.019*	
N5	0.2204 (2)	0.89140 (11)	0.79258 (15)	0.0241 (7)	
H5A	0.2105	0.8591	0.7770	0.029*	
H5B	0.2635	0.8902	0.8308	0.029*	
N6	0.3308 (3)	1.01571 (12)	0.63761 (16)	0.0309 (8)	
H6A	0.3844	1.0377	0.6537	0.037*	
H6B	0.3495	1.0054	0.5959	0.037*	
N7	0.6399 (2)	0.53819 (10)	0.48058 (14)	0.0178 (6)	
N8	0.5834 (2)	0.46113 (11)	0.56943 (13)	0.0177 (6)	
N9	0.4580 (2)	0.55280 (10)	0.56899 (13)	0.0165 (6)	
H9A	0.3827	0.5517	0.5754	0.025*	
H9B	0.4774	0.5840	0.5545	0.025*	
H9C	0.4953	0.5461	0.6077	0.025*	
N10	0.4716 (3)	0.73221 (11)	0.70742 (14)	0.0218 (6)	
N11	0.1892 (2)	0.65613 (11)	0.74405 (14)	0.0193 (6)	
N12	0.2663 (2)	0.72071 (11)	0.64291 (14)	0.0231 (7)	
N13	0.4026 (2)	0.66793 (11)	0.81050 (14)	0.0220 (7)	
N14	0.3879 (2)	0.63442 (11)	0.67497 (14)	0.0207 (6)	
H14A	0.4369	0.6162	0.7004	0.031*	
H14B	0.4232	0.6457	0.6385	0.031*	
H14C	0.3288	0.6146	0.6625	0.031*	
N15	0.2674 (2)	0.75427 (10)	0.77962 (13)	0.0177 (6)	
H15A	0.2317	0.7426	0.8158	0.027*	
H15B	0.2178	0.7716	0.7534	0.027*	
H15C	0.3245	0.7751	0.7925	0.027*	
N16	0.392 (2)	1.0079 (9)	0.4612 (10)	0.114 (9)	0.50
C1	0.4527 (3)	0.88644 (16)	0.63489 (19)	0.0324 (9)	
H1A	0.5152	0.8623	0.6295	0.039*	
H1B	0.4630	0.9148	0.6038	0.039*	
C2	0.3426 (4)	0.86090 (16)	0.6210 (2)	0.0373 (11)	
H2C	0.3376	0.8505	0.5741	0.045*	
H2D	0.3362	0.8304	0.6487	0.045*	
C3	0.5415 (3)	0.93959 (16)	0.72333 (19)	0.0306 (9)	
H3C	0.5485	0.9670	0.6906	0.037*	
H3D	0.6139	0.9213	0.7262	0.037*	
C4	0.5138 (3)	0.96112 (15)	0.7895 (2)	0.0314 (9)	
H4A	0.5184	0.9345	0.8236	0.038*	
H4B	0.5679	0.9881	0.8016	0.038*	
C5	0.1172 (3)	0.97132 (13)	0.79187 (17)	0.0192 (7)	
H5C	0.0417	0.9865	0.7957	0.023*	
H5D	0.1681	0.9875	0.8248	0.023*	
C6	0.1115 (3)	0.91536 (13)	0.80462 (17)	0.0199 (7)	
H6C	0.0902	0.9094	0.8509	0.024*	
H6D	0.0532	0.9001	0.7754	0.024*	
C7	0.1692 (3)	1.03318 (13)	0.70640 (18)	0.0239 (8)	
H7A	0.2179	1.0509	0.7391	0.029*	
H7B	0.0940	1.0491	0.7065	0.029*	
C8	0.2187 (3)	1.03785 (15)	0.63842 (19)	0.0321 (9)	
H8A	0.1694	1.0205	0.6057	0.038*	
H8B	0.2229	1.0739	0.6259	0.038*	
C9	0.7253 (3)	0.55551 (13)	0.46626 (16)	0.0173 (7)	
C10	0.6314 (3)	0.43966 (14)	0.61159 (17)	0.0207 (8)	
C11	0.5507 (3)	0.75268 (14)	0.68755 (17)	0.0232 (8)	
C12	0.1079 (3)	0.63266 (14)	0.75061 (17)	0.0227 (8)	
C13	0.2537 (3)	0.73456 (14)	0.58939 (18)	0.0229 (8)	
C14	0.4489 (4)	0.65331 (15)	0.8572 (2)	0.0323 (9)	
C15	0.1477 (3)	0.56222 (17)	0.48878 (18)	0.0347 (10)	
H15D	0.1785	0.5289	0.4787	0.052*	
H15E	0.2047	0.5881	0.4809	0.052*	
H15F	0.0816	0.5687	0.4604	0.052*	
C16	0.0651 (3)	0.62831 (14)	0.5749 (2)	0.0320 (9)	
H16A	0.0479	0.6380	0.6202	0.048*	
H16B	−0.0019	0.6327	0.5466	0.048*	
H16C	0.1257	0.6497	0.5587	0.048*	
C17	0.6303 (3)	0.78206 (17)	0.84638 (19)	0.0354 (10)	
H17A	0.6714	0.7976	0.8103	0.053*	
H17B	0.6825	0.7747	0.8831	0.053*	
H17C	0.5953	0.7507	0.8308	0.053*	
C18	0.4587 (3)	0.78282 (16)	0.9293 (2)	0.0333 (9)	
H18A	0.3940	0.7998	0.9484	0.050*	
H18B	0.4333	0.7525	0.9058	0.050*	
H18C	0.5122	0.7734	0.9646	0.050*	
C19	0.6980 (4)	0.64204 (19)	0.7345 (4)	0.100 (3)	
H19A	0.6636	0.6468	0.7775	0.150*	
H19B	0.6548	0.6606	0.7008	0.150*	
H19C	0.7753	0.6547	0.7364	0.150*	
C20	0.7537 (4)	0.5805 (3)	0.6345 (2)	0.088 (2)	
H20A	0.7405	0.5483	0.6120	0.132*	
H20B	0.8346	0.5871	0.6377	0.132*	
H20C	0.7168	0.6076	0.6094	0.132*	
C21	−0.0235 (5)	0.6549 (2)	0.9361 (3)	0.092 (2)	
H21A	−0.0739	0.6619	0.8983	0.138*	
H21B	−0.0366	0.6795	0.9710	0.138*	
H21C	−0.0380	0.6209	0.9527	0.138*	
C22	0.1829 (6)	0.6484 (2)	0.9897 (3)	0.087 (2)	
H22A	0.2644	0.6527	0.9868	0.131*	
H22B	0.1663	0.6140	1.0042	0.131*	
H22C	0.1534	0.6725	1.0215	0.131*	
C23A	−0.0318 (5)	0.7965 (3)	0.6057 (3)	0.0453 (19)	0.65
H23A	−0.0061	0.8266	0.5824	0.068*	0.65
H23B	−0.1106	0.7899	0.5936	0.068*	0.65
H23C	0.0141	0.7676	0.5932	0.068*	0.65
C24A	−0.0427 (6)	0.7473 (3)	0.7218 (5)	0.067 (3)	0.65
H24A	−0.0237	0.7466	0.7691	0.101*	0.65
H24B	0.0038	0.7228	0.6987	0.101*	0.65
H24C	−0.1222	0.7387	0.7151	0.101*	0.65
C23B	−0.0229 (13)	0.7469 (6)	0.6427 (8)	0.061 (4)*	0.35
H23D	−0.0018	0.7550	0.5975	0.092*	0.35
H23E	−0.0989	0.7328	0.6424	0.092*	0.35
H23F	0.0300	0.7222	0.6615	0.092*	0.35
C24B	−0.0647 (11)	0.7730 (7)	0.7657 (7)	0.057 (5)	0.35
H24D	−0.0593	0.7958	0.8037	0.086*	0.35
H24E	−0.0169	0.7435	0.7737	0.086*	0.35
H24F	−0.1428	0.7621	0.7592	0.086*	0.35
C25	0.6848 (5)	0.70103 (19)	0.5048 (3)	0.073 (2)	
H25A	0.7251	0.7078	0.5465	0.109*	
H25B	0.7061	0.6677	0.4884	0.109*	
H25C	0.7044	0.7267	0.4723	0.109*	
C26	0.4929 (6)	0.6836 (2)	0.4377 (3)	0.084 (2)	
H26A	0.4115	0.6782	0.4372	0.127*	
H26B	0.5113	0.7099	0.4057	0.127*	
H26C	0.5308	0.6522	0.4259	0.127*	
C27A	−0.0992 (8)	0.9734 (5)	0.5386 (4)	0.106 (5)	0.687 (10)
H27A	−0.0570	0.9544	0.5058	0.159*	0.687 (10)
H27B	−0.0600	1.0049	0.5490	0.159*	0.687 (10)
H27C	−0.1745	0.9809	0.5208	0.159*	0.687 (10)
C27B	−0.1905 (12)	0.9106 (6)	0.6212 (9)	0.053 (5)	0.313 (10)
H27D	−0.1720	0.8783	0.6007	0.080*	0.313 (10)
H27E	−0.2684	0.9196	0.6100	0.080*	0.313 (10)
H27F	−0.1812	0.9078	0.6692	0.080*	0.313 (10)
C28	−0.1568 (5)	0.9918 (3)	0.6584 (3)	0.095 (3)	
H28A	−0.1576	1.0219	0.6303	0.142*	0.687 (10)
H28B	−0.1049	0.9970	0.6959	0.142*	0.687 (10)
H28C	−0.2325	0.9856	0.6745	0.142*	0.687 (10)
H28D	−0.1834	1.0232	0.6403	0.142*	0.313 (10)
H28E	−0.0996	0.9983	0.6914	0.142*	0.313 (10)
H28F	−0.2180	0.9741	0.6779	0.142*	0.313 (10)
C29	0.4824 (13)	1.0064 (6)	0.4869 (6)	0.051 (3)	0.50
O1W	0.2435 (10)	0.9301 (5)	0.4986 (5)	0.135 (4)	0.50
H1W	0.2951	0.9471	0.4799	0.202*	0.50
H2W	0.2122	0.9122	0.4672	0.202*	0.50

### Atomic displacement parameters (Å^2^)

**Table:**

	*U*^11^	*U*^22^	*U*^33^	*U*^12^	*U*^13^	*U*^23^
Cu1	0.0146 (2)	0.0202 (2)	0.0239 (2)	0.00023 (18)	0.00210 (17)	−0.00534 (18)
Cr1	0.0117 (4)	0.0145 (4)	0.0144 (4)	−0.0017 (3)	0.0034 (3)	−0.0010 (3)
Cr2	0.0186 (3)	0.0144 (3)	0.0175 (3)	0.0012 (2)	0.0004 (2)	−0.0031 (2)
S1	0.0165 (5)	0.0333 (5)	0.0317 (5)	−0.0098 (4)	0.0046 (4)	−0.0028 (4)
S2	0.0243 (5)	0.0695 (8)	0.0308 (6)	0.0092 (5)	−0.0032 (4)	0.0179 (5)
S3	0.0208 (5)	0.0527 (7)	0.0474 (7)	−0.0039 (5)	0.0040 (5)	0.0156 (5)
S4	0.0177 (5)	0.0299 (5)	0.0484 (7)	−0.0035 (4)	0.0016 (4)	0.0044 (5)
S5	0.0626 (8)	0.0722 (9)	0.0218 (6)	−0.0335 (7)	−0.0134 (5)	0.0117 (5)
S6	0.0948 (12)	0.0582 (9)	0.0562 (9)	−0.0036 (8)	−0.0448 (8)	0.0185 (7)
S7	0.0139 (4)	0.0208 (4)	0.0216 (4)	0.0001 (3)	0.0000 (3)	0.0017 (3)
S8	0.0223 (5)	0.0233 (5)	0.0260 (5)	−0.0003 (4)	−0.0066 (4)	−0.0056 (4)
S9	0.0180 (4)	0.0274 (5)	0.0273 (5)	0.0008 (4)	−0.0030 (4)	0.0007 (4)
S10	0.0944 (11)	0.0314 (6)	0.0358 (7)	−0.0011 (6)	0.0315 (7)	−0.0065 (5)
S11	0.0220 (5)	0.0359 (6)	0.0429 (6)	0.0041 (4)	−0.0056 (4)	−0.0031 (5)
S12	0.0632 (8)	0.0174 (5)	0.0487 (7)	−0.0048 (5)	0.0359 (6)	−0.0038 (5)
S13A	0.0429 (16)	0.063 (2)	0.071 (2)	0.0104 (17)	−0.0320 (15)	−0.0288 (17)
S13B	0.039 (2)	0.041 (3)	0.032 (3)	0.007 (2)	0.0019 (19)	−0.005 (2)
S14	0.059 (3)	0.117 (4)	0.046 (2)	0.031 (3)	−0.009 (2)	−0.010 (2)
O1	0.0159 (12)	0.0334 (15)	0.0271 (14)	0.0012 (11)	−0.0061 (10)	0.0061 (11)
O2	0.0298 (15)	0.0218 (14)	0.0381 (16)	0.0051 (12)	−0.0125 (12)	−0.0027 (12)
O3	0.0160 (12)	0.0299 (15)	0.0384 (16)	−0.0035 (11)	0.0046 (11)	0.0002 (12)
O4	0.111 (3)	0.0321 (18)	0.049 (2)	0.001 (2)	0.048 (2)	−0.0074 (15)
O5	0.0248 (15)	0.0285 (15)	0.0467 (17)	−0.0006 (12)	−0.0103 (13)	−0.0022 (13)
O6	0.093 (3)	0.0193 (15)	0.051 (2)	−0.0100 (16)	0.0495 (19)	−0.0043 (14)
O7	0.0366 (19)	0.093 (3)	0.082 (3)	0.016 (2)	−0.0264 (18)	−0.048 (2)
N1	0.0202 (16)	0.0220 (17)	0.0349 (19)	0.0049 (13)	0.0060 (14)	−0.0003 (14)
N2	0.0299 (18)	0.0311 (19)	0.0318 (19)	0.0000 (15)	0.0010 (15)	−0.0044 (15)
N3	0.0216 (16)	0.0242 (17)	0.0307 (18)	−0.0045 (13)	0.0027 (13)	0.0011 (14)
N4	0.0154 (14)	0.0153 (14)	0.0177 (15)	0.0026 (11)	−0.0010 (11)	−0.0015 (11)
N5	0.0233 (16)	0.0169 (15)	0.0320 (18)	0.0022 (13)	−0.0042 (14)	−0.0003 (13)
N6	0.0262 (18)	0.0312 (19)	0.0356 (19)	−0.0032 (15)	0.0086 (15)	0.0006 (15)
N7	0.0153 (15)	0.0177 (15)	0.0206 (15)	−0.0043 (12)	0.0053 (12)	0.0000 (12)
N8	0.0147 (14)	0.0248 (16)	0.0137 (14)	−0.0027 (12)	0.0023 (12)	−0.0039 (12)
N9	0.0158 (14)	0.0174 (15)	0.0166 (15)	−0.0021 (12)	0.0038 (11)	−0.0017 (11)
N10	0.0263 (16)	0.0189 (16)	0.0199 (16)	0.0012 (13)	−0.0030 (13)	−0.0009 (12)
N11	0.0183 (15)	0.0208 (16)	0.0192 (15)	−0.0003 (12)	0.0069 (12)	−0.0050 (12)
N12	0.0241 (16)	0.0236 (17)	0.0214 (17)	−0.0008 (13)	−0.0030 (13)	−0.0026 (13)
N13	0.0259 (17)	0.0210 (16)	0.0190 (16)	0.0011 (13)	−0.0035 (13)	0.0000 (12)
N14	0.0228 (16)	0.0175 (15)	0.0220 (16)	0.0013 (13)	0.0021 (12)	−0.0036 (12)
N15	0.0203 (15)	0.0158 (14)	0.0169 (15)	0.0033 (12)	−0.0004 (12)	−0.0030 (11)
N16	0.057 (9)	0.17 (2)	0.112 (14)	0.046 (13)	−0.032 (8)	0.009 (12)
C1	0.035 (2)	0.032 (2)	0.030 (2)	0.0081 (19)	0.0111 (18)	−0.0002 (17)
C2	0.052 (3)	0.030 (2)	0.031 (2)	0.001 (2)	0.013 (2)	−0.0166 (18)
C3	0.0195 (19)	0.037 (2)	0.035 (2)	0.0050 (17)	0.0014 (16)	0.0052 (18)
C4	0.0170 (19)	0.028 (2)	0.049 (3)	−0.0006 (16)	−0.0048 (17)	0.0008 (19)
C5	0.0107 (16)	0.0172 (17)	0.030 (2)	0.0013 (14)	0.0071 (14)	−0.0027 (15)
C6	0.0189 (18)	0.0231 (19)	0.0181 (18)	−0.0018 (15)	0.0063 (14)	0.0030 (14)
C7	0.0238 (19)	0.0178 (18)	0.030 (2)	0.0031 (15)	0.0038 (16)	0.0015 (15)
C8	0.040 (2)	0.026 (2)	0.030 (2)	0.0015 (18)	−0.0015 (18)	0.0167 (17)
C9	0.0210 (18)	0.0167 (17)	0.0138 (17)	0.0050 (15)	−0.0032 (14)	−0.0043 (13)
C10	0.0174 (17)	0.0264 (19)	0.0185 (19)	−0.0066 (15)	0.0063 (14)	−0.0065 (15)
C11	0.025 (2)	0.025 (2)	0.0201 (19)	0.0000 (16)	−0.0038 (15)	0.0050 (15)
C12	0.024 (2)	0.0211 (19)	0.023 (2)	0.0090 (16)	0.0012 (15)	−0.0034 (15)
C13	0.0180 (18)	0.026 (2)	0.024 (2)	−0.0084 (15)	−0.0009 (15)	−0.0051 (16)
C14	0.038 (2)	0.023 (2)	0.036 (2)	−0.0041 (18)	−0.0071 (19)	−0.0039 (17)
C15	0.028 (2)	0.056 (3)	0.019 (2)	0.016 (2)	0.0031 (16)	0.0029 (18)
C16	0.038 (2)	0.021 (2)	0.037 (2)	0.0070 (18)	−0.0115 (18)	−0.0097 (17)
C17	0.030 (2)	0.053 (3)	0.024 (2)	0.018 (2)	0.0010 (17)	−0.0004 (19)
C18	0.025 (2)	0.037 (2)	0.038 (2)	0.0025 (18)	−0.0002 (18)	−0.0002 (19)
C19	0.030 (3)	0.030 (3)	0.240 (9)	0.004 (2)	−0.011 (4)	−0.045 (4)
C20	0.031 (3)	0.192 (8)	0.041 (3)	−0.045 (4)	0.006 (2)	0.010 (4)
C21	0.104 (5)	0.073 (5)	0.102 (5)	−0.010 (4)	0.067 (4)	−0.012 (4)
C22	0.158 (7)	0.064 (4)	0.039 (3)	−0.013 (4)	0.009 (4)	0.007 (3)
C23A	0.029 (4)	0.071 (5)	0.036 (4)	−0.006 (4)	−0.008 (3)	−0.020 (4)
C24A	0.027 (4)	0.059 (6)	0.113 (8)	−0.019 (4)	−0.029 (5)	0.040 (5)
C24B	0.030 (7)	0.085 (12)	0.057 (10)	0.009 (8)	0.011 (7)	−0.034 (9)
C25	0.075 (4)	0.040 (3)	0.106 (5)	0.022 (3)	0.065 (4)	0.025 (3)
C26	0.150 (7)	0.052 (4)	0.053 (4)	−0.032 (4)	0.041 (4)	−0.018 (3)
C27A	0.097 (8)	0.193 (13)	0.028 (5)	0.084 (8)	−0.003 (4)	0.003 (6)
C27B	0.018 (8)	0.050 (10)	0.091 (13)	−0.014 (7)	−0.018 (8)	0.012 (9)
C28	0.057 (4)	0.154 (7)	0.072 (4)	0.039 (4)	−0.019 (3)	−0.065 (4)
C29	0.056 (10)	0.070 (10)	0.026 (8)	0.002 (7)	0.007 (7)	0.006 (6)
O1W	0.173 (12)	0.150 (11)	0.079 (7)	−0.038 (9)	−0.030 (7)	0.028 (7)

### Geometric parameters (Å, °)

**Table:**

Cu1—N1	2.016 (3)	N14—H14C	0.9101
Cu1—N4	2.018 (3)	N15—H15A	0.9101
Cu1—N2	2.079 (3)	N15—H15B	0.9099
Cu1—N3	2.100 (3)	N15—H15C	0.9099
Cu1—N5	2.295 (3)	N16—C29	1.19 (2)
Cu1—N6	2.553 (3)	C1—C2	1.497 (6)
Cr1—N8^i^	1.993 (3)	C1—H1A	0.9900
Cr1—N8	1.993 (3)	C1—H1B	0.9900
Cr1—N7	1.997 (3)	C2—H2C	0.9900
Cr1—N7^i^	1.997 (3)	C2—H2D	0.9900
Cr1—N9	2.049 (3)	C3—C4	1.501 (6)
Cr1—N9^i^	2.049 (3)	C3—H3C	0.9900
Cr2—N13	1.993 (3)	C3—H3D	0.9900
Cr2—N12	1.996 (3)	C4—H4A	0.9900
Cr2—N10	2.000 (3)	C4—H4B	0.9900
Cr2—N11	2.007 (3)	C5—C6	1.509 (5)
Cr2—N14	2.042 (3)	C5—H5C	0.9900
Cr2—N15	2.059 (3)	C5—H5D	0.9900
S1—C9	1.634 (4)	C6—H6C	0.9900
S2—C10	1.618 (4)	C6—H6D	0.9900
S3—C11	1.627 (4)	C7—C8	1.516 (5)
S4—C12	1.637 (4)	C7—H7A	0.9900
S5—C13	1.626 (4)	C7—H7B	0.9900
S6—C14	1.626 (4)	C8—H8A	0.9900
S7—O1	1.513 (2)	C8—H8B	0.9900
S7—C16	1.774 (4)	C15—H15D	0.9800
S7—C15	1.788 (4)	C15—H15E	0.9801
S8—O2	1.512 (3)	C15—H15F	0.9800
S8—C18	1.774 (4)	C16—H16A	0.9798
S8—C17	1.775 (4)	C16—H16B	0.9800
S9—O3	1.505 (3)	C16—H16C	0.9801
S9—C19	1.752 (5)	C17—H17A	0.9800
S9—C20	1.773 (5)	C17—H17B	0.9800
S10—O4	1.501 (3)	C17—H17C	0.9800
S10—C22	1.773 (6)	C18—H18A	0.9800
S10—C21	1.787 (6)	C18—H18B	0.9800
S11—O5	1.505 (3)	C18—H18C	0.9799
S11—C24A	1.720 (8)	C19—H19A	0.9798
S11—C23A	1.771 (6)	C19—H19B	0.9800
S11—C24B	1.840 (16)	C19—H19C	0.9800
S11—C23B	1.878 (15)	C20—H20A	0.9799
S12—O6	1.517 (3)	C20—H20B	0.9800
S12—C25	1.770 (5)	C20—H20C	0.9799
S12—C26	1.774 (6)	C21—H21A	0.9800
S13A—O7	1.488 (4)	C21—H21B	0.9801
S13A—C27A	1.749 (12)	C21—H21C	0.9799
S13A—C28	1.835 (6)	C22—H22A	0.9800
S13A—H27D	1.7359	C22—H22B	0.9801
S13A—H27F	1.6703	C22—H22C	0.9799
S13B—O7	1.680 (8)	C23A—H23A	0.9800
S13B—C28	1.769 (8)	C23A—H23B	0.9800
S13B—C27B	1.780 (16)	C23A—H23C	0.9801
S14—C29	1.599 (16)	C23A—H23D	1.1714
N1—C3	1.457 (5)	C24A—H24A	0.9798
N1—C1	1.468 (5)	C24A—H24B	0.9800
N1—H1N	0.9718	C24A—H24C	0.9800
N2—C2	1.493 (5)	C23B—H23D	0.9800
N2—H2A	0.9200	C23B—H23E	0.9801
N2—H2B	0.9200	C23B—H23F	0.9801
N3—C4	1.472 (5)	C24B—H24D	0.9798
N3—H3A	0.9200	C24B—H24E	0.9800
N3—H3B	0.9200	C24B—H24F	0.9801
N4—C7	1.475 (4)	C25—H25A	0.9800
N4—C5	1.481 (4)	C25—H25B	0.9800
N4—H4N	0.9853	C25—H25C	0.9801
N5—C6	1.470 (4)	C26—H26A	0.9800
N5—H5A	0.9200	C26—H26B	0.9798
N5—H5B	0.9200	C26—H26C	0.9801
N6—C8	1.459 (5)	C27A—H27A	0.9800
N6—H6A	0.9200	C27A—H27B	0.9801
N6—H6B	0.9200	C27A—H27C	0.9801
N7—C9	1.158 (4)	C27B—H27D	0.9799
N8—C10	1.167 (4)	C27B—H27E	0.9800
N9—H9A	0.9099	C27B—H27F	0.9801
N9—H9B	0.9099	C28—H28A	0.9800
N9—H9C	0.9099	C28—H28B	0.9800
N10—C11	1.167 (4)	C28—H28C	0.9800
N11—C12	1.162 (4)	C28—H28D	0.9603
N12—C13	1.152 (4)	C28—H28E	0.9600
N13—C14	1.153 (5)	C28—H28F	0.9601
N14—H14A	0.9100	O1W—H1W	0.8575
N14—H14B	0.9099	O1W—H2W	0.8710
			
N1—Cu1—N4	177.20 (12)	C4—C3—H3C	110.0
N1—Cu1—N2	84.23 (13)	N1—C3—H3D	110.0
N4—Cu1—N2	96.26 (12)	C4—C3—H3D	110.0
N1—Cu1—N3	82.28 (12)	H3C—C3—H3D	108.4
N4—Cu1—N3	96.95 (12)	N3—C4—C3	108.1 (3)
N2—Cu1—N3	165.45 (12)	N3—C4—H4A	110.1
N1—Cu1—N5	101.97 (12)	C3—C4—H4A	110.1
N4—Cu1—N5	80.75 (11)	N3—C4—H4B	110.1
N2—Cu1—N5	94.29 (12)	C3—C4—H4B	110.1
N3—Cu1—N5	93.81 (11)	H4A—C4—H4B	108.4
N1—Cu1—N6	99.85 (12)	N4—C5—C6	108.7 (3)
N4—Cu1—N6	77.40 (10)	N4—C5—H5C	110.0
N2—Cu1—N6	90.22 (12)	C6—C5—H5C	110.0
N3—Cu1—N6	86.75 (12)	N4—C5—H5D	109.9
N5—Cu1—N6	158.05 (10)	C6—C5—H5D	109.9
N8^i^—Cr1—N8	180.00 (11)	H5C—C5—H5D	108.3
N8^i^—Cr1—N7	90.19 (11)	N5—C6—C5	110.7 (3)
N8—Cr1—N7	89.81 (11)	N5—C6—H6C	109.5
N8^i^—Cr1—N7^i^	89.81 (11)	C5—C6—H6C	109.5
N8—Cr1—N7^i^	90.19 (11)	N5—C6—H6D	109.5
N7—Cr1—N7^i^	180.00 (14)	C5—C6—H6D	109.5
N8^i^—Cr1—N9	90.18 (11)	H6C—C6—H6D	108.1
N8—Cr1—N9	89.82 (11)	N4—C7—C8	109.9 (3)
N7—Cr1—N9	90.27 (11)	N4—C7—H7A	109.7
N7^i^—Cr1—N9	89.73 (11)	C8—C7—H7A	109.7
N8^i^—Cr1—N9^i^	89.82 (11)	N4—C7—H7B	109.7
N8—Cr1—N9^i^	90.18 (11)	C8—C7—H7B	109.7
N7—Cr1—N9^i^	89.73 (11)	H7A—C7—H7B	108.2
N7^i^—Cr1—N9^i^	90.27 (11)	N6—C8—C7	110.6 (3)
N9—Cr1—N9^i^	180.00 (11)	N6—C8—H8A	109.5
N13—Cr2—N12	177.44 (12)	C7—C8—H8A	109.5
N13—Cr2—N10	90.40 (12)	N6—C8—H8B	109.5
N12—Cr2—N10	87.57 (12)	C7—C8—H8B	109.5
N13—Cr2—N11	91.60 (12)	H8A—C8—H8B	108.1
N12—Cr2—N11	90.40 (12)	N7—C9—S1	179.1 (4)
N10—Cr2—N11	177.59 (12)	N8—C10—S2	178.8 (3)
N13—Cr2—N14	91.47 (12)	N10—C11—S3	178.2 (3)
N12—Cr2—N14	86.96 (12)	N11—C12—S4	179.5 (4)
N10—Cr2—N14	89.72 (12)	N12—C13—S5	178.7 (3)
N11—Cr2—N14	88.88 (11)	N13—C14—S6	178.8 (4)
N13—Cr2—N15	89.86 (11)	S7—C15—H15D	109.6
N12—Cr2—N15	91.77 (12)	S7—C15—H15E	109.5
N10—Cr2—N15	92.23 (12)	H15D—C15—H15E	109.5
N11—Cr2—N15	89.12 (11)	S7—C15—H15F	109.3
N14—Cr2—N15	177.62 (12)	H15D—C15—H15F	109.5
O1—S7—C16	106.46 (17)	H15E—C15—H15F	109.5
O1—S7—C15	106.01 (17)	S7—C16—H16A	109.5
C16—S7—C15	97.4 (2)	S7—C16—H16B	109.5
O2—S8—C18	106.05 (18)	H16A—C16—H16B	109.5
O2—S8—C17	106.28 (17)	S7—C16—H16C	109.5
C18—S8—C17	97.8 (2)	H16A—C16—H16C	109.5
O3—S9—C19	106.2 (2)	H16B—C16—H16C	109.5
O3—S9—C20	105.6 (2)	S8—C17—H17A	109.4
C19—S9—C20	99.9 (4)	S8—C17—H17B	109.5
O4—S10—C22	107.0 (3)	H17A—C17—H17B	109.5
O4—S10—C21	105.6 (3)	S8—C17—H17C	109.5
C22—S10—C21	97.3 (3)	H17A—C17—H17C	109.5
O5—S11—C24A	105.8 (3)	H17B—C17—H17C	109.5
O5—S11—C23A	104.2 (2)	S8—C18—H18A	109.4
C24A—S11—C23A	101.2 (5)	S8—C18—H18B	109.5
O5—S11—C24B	105.2 (5)	H18A—C18—H18B	109.5
O5—S11—C23B	104.2 (5)	S8—C18—H18C	109.5
C24B—S11—C23B	90.7 (7)	H18A—C18—H18C	109.5
O6—S12—C25	104.4 (2)	H18B—C18—H18C	109.5
O6—S12—C26	105.3 (2)	S9—C19—H19A	109.3
C25—S12—C26	98.0 (3)	S9—C19—H19B	109.5
O7—S13A—C27A	100.8 (5)	H19A—C19—H19B	109.5
O7—S13A—C28	105.9 (3)	S9—C19—H19C	109.6
C27A—S13A—C28	92.2 (5)	H19A—C19—H19C	109.5
O7—S13B—C28	100.9 (4)	H19B—C19—H19C	109.5
O7—S13B—C27B	89.7 (6)	S9—C20—H20A	109.5
C28—S13B—C27B	81.4 (7)	S9—C20—H20B	109.3
C3—N1—C1	117.3 (3)	H20A—C20—H20B	109.5
C3—N1—Cu1	107.9 (2)	S9—C20—H20C	109.6
C1—N1—Cu1	107.2 (2)	H20A—C20—H20C	109.5
C3—N1—H1N	106.6	H20B—C20—H20C	109.5
C1—N1—H1N	106.0	S10—C21—H21A	109.7
Cu1—N1—H1N	111.8	S10—C21—H21B	109.4
C2—N2—Cu1	107.2 (2)	H21A—C21—H21B	109.5
C2—N2—H2A	110.3	S10—C21—H21C	109.3
Cu1—N2—H2A	110.3	H21A—C21—H21C	109.5
C2—N2—H2B	110.3	H21B—C21—H21C	109.5
Cu1—N2—H2B	110.3	S10—C22—H22A	109.4
H2A—N2—H2B	108.5	S10—C22—H22B	109.4
C4—N3—Cu1	109.5 (2)	H22A—C22—H22B	109.5
C4—N3—H3A	109.8	S10—C22—H22C	109.5
Cu1—N3—H3A	109.8	H22A—C22—H22C	109.5
C4—N3—H3B	109.8	H22B—C22—H22C	109.5
Cu1—N3—H3B	109.8	S11—C23A—H23A	109.0
H3A—N3—H3B	108.2	S11—C23A—H23B	109.5
C7—N4—C5	113.7 (3)	H23A—C23A—H23B	109.5
C7—N4—Cu1	113.3 (2)	S11—C23A—H23C	109.9
C5—N4—Cu1	108.8 (2)	H23A—C23A—H23C	109.5
C7—N4—H4N	107.6	H23B—C23A—H23C	109.5
C5—N4—H4N	102.6	S11—C23A—H23D	105.4
Cu1—N4—H4N	110.2	S11—C24A—H24A	109.1
C6—N5—Cu1	105.4 (2)	S11—C24A—H24B	110.0
C6—N5—H5A	110.7	H24A—C24A—H24B	109.5
Cu1—N5—H5A	110.7	S11—C24A—H24C	109.3
C6—N5—H5B	110.7	H24A—C24A—H24C	109.5
Cu1—N5—H5B	110.7	H24B—C24A—H24C	109.5
H5A—N5—H5B	108.8	S11—C23B—H23D	107.8
C8—N6—Cu1	100.4 (2)	S11—C23B—H23E	109.9
C8—N6—H6A	111.7	H23D—C23B—H23E	109.5
Cu1—N6—H6A	111.7	S11—C23B—H23F	110.7
C8—N6—H6B	111.7	H23D—C23B—H23F	109.5
Cu1—N6—H6B	111.7	H23E—C23B—H23F	109.5
H6A—N6—H6B	109.5	S11—C24B—H24D	108.8
C9—N7—Cr1	172.5 (3)	S11—C24B—H24E	110.2
C10—N8—Cr1	177.7 (3)	H24D—C24B—H24E	109.5
Cr1—N9—H9A	109.4	S11—C24B—H24F	109.4
Cr1—N9—H9B	109.5	H24D—C24B—H24F	109.5
H9A—N9—H9B	109.5	H24E—C24B—H24F	109.5
Cr1—N9—H9C	109.5	S12—C25—H25A	109.4
H9A—N9—H9C	109.5	S12—C25—H25B	109.7
H9B—N9—H9C	109.5	H25A—C25—H25B	109.5
C11—N10—Cr2	171.6 (3)	S12—C25—H25C	109.3
C12—N11—Cr2	176.6 (3)	H25A—C25—H25C	109.5
C13—N12—Cr2	164.3 (3)	H25B—C25—H25C	109.5
C14—N13—Cr2	176.6 (3)	S12—C26—H26A	109.5
Cr2—N14—H14A	109.5	S12—C26—H26B	109.3
Cr2—N14—H14B	109.4	H26A—C26—H26B	109.5
H14A—N14—H14B	109.5	S12—C26—H26C	109.6
Cr2—N14—H14C	109.5	H26A—C26—H26C	109.5
H14A—N14—H14C	109.5	H26B—C26—H26C	109.5
H14B—N14—H14C	109.5	S13A—C27A—H27A	109.5
Cr2—N15—H15A	109.5	S13A—C27A—H27B	109.6
Cr2—N15—H15B	109.5	H27A—C27A—H27B	109.5
H15A—N15—H15B	109.5	S13A—C27A—H27C	109.3
Cr2—N15—H15C	109.4	H27A—C27A—H27C	109.5
H15A—N15—H15C	109.5	H27B—C27A—H27C	109.5
H15B—N15—H15C	109.5	S13B—C27B—H27D	109.6
S14^ii^—N16—C29	69 (3)	S13B—C27B—H27E	109.0
S14^ii^—N16—C29^ii^	57 (2)	H27D—C27B—H27E	109.5
C29—N16—C29^ii^	13.6 (12)	S13B—C27B—H27F	109.8
N1—C1—C2	107.9 (3)	H27D—C27B—H27F	109.5
N1—C1—H1A	110.1	H27E—C27B—H27F	109.5
C2—C1—H1A	110.1	S13A—C28—H28A	109.5
N1—C1—H1B	110.1	S13A—C28—H28B	109.5
C2—C1—H1B	110.1	H28A—C28—H28B	109.5
H1A—C1—H1B	108.4	S13A—C28—H28C	109.5
N2—C2—C1	108.7 (3)	H28A—C28—H28C	109.5
N2—C2—H2C	109.9	H28B—C28—H28C	109.5
C1—C2—H2C	109.9	S13B—C28—H28E	110.4
N2—C2—H2D	109.9	H28D—C28—H28E	109.5
C1—C2—H2D	109.9	H28D—C28—H28F	109.5
H2C—C2—H2D	108.3	H28E—C28—H28F	109.5
N1—C3—C4	108.5 (3)	N16—C29—S14	170.5 (19)
N1—C3—H3C	110.0	H1W—O1W—H2W	105.4

Symmetry codes: (i) −*x*+1, −*y*+1, −*z*+1; (ii) −*x*+1, −*y*+2, −*z*+1.

### Hydrogen-bond geometry (Å, °)

**Table:**

*D*—H···*A*	*D*—H	H···*A*	*D*···*A*	*D*—H···*A*
O1W—H1W···N16	0.86	2.02	2.83 (3)	157
N1—H1N···O2	0.97	2.10	3.029 (4)	158
N2—H2A···O5	0.92	2.26	3.083 (4)	149
N2—H2B···O1W	0.92	2.09	2.919 (10)	150
N4—H4N···O7	0.99	1.96	2.928 (4)	167
N5—H5A···O5	0.92	2.24	2.996 (4)	140
N6—H6B···S14^ii^	0.92	2.62	3.539 (6)	174
N9—H9A···O1	0.91	2.16	3.049 (3)	165
N9—H9B···O6	0.91	2.02	2.881 (4)	157
N9—H9C···O3	0.91	2.23	3.093 (4)	157
N14—H14A···O3	0.91	2.19	3.001 (4)	148
N14—H14B···O6	0.91	1.96	2.853 (4)	169
N14—H14C···O1	0.91	2.18	3.066 (4)	166
N15—H15A···O4	0.91	2.12	3.023 (4)	175
N15—H15B···O5	0.91	2.06	2.965 (4)	173
N15—H15C···O2	0.91	2.09	2.992 (4)	172
O1W—H1W···N16	0.86	2.02	2.83 (3)	157
N3—H3A···S4^iii^	0.92	2.67	3.518 (3)	154
N5—H5B···S1^iv^	0.92	2.66	3.529 (3)	158
O1W—H2W···S6^v^	0.87	2.77	3.523 (11)	145
N6—H6A···S4^iii^	0.92	2.80	3.686 (4)	161

Symmetry codes: (ii) −*x*+1, −*y*+2, −*z*+1; (iii) −*x*+1/2, *y*+1/2, −*z*+3/2; (iv) *x*−1/2, −*y*+3/2, *z*+1/2; (v) *x*−1/2, −*y*+3/2, *z*−1/2.
